# LTV-MPC Approach for Automated Vehicle Path Following at the Limit of Handling

**DOI:** 10.3390/s22155807

**Published:** 2022-08-03

**Authors:** Ádám Domina, Viktor Tihanyi

**Affiliations:** Department of Automotive Technologies, Budapest University of Technology and Economics, 1111 Budapest, Hungary; tihanyi.viktor@kjk.bme.hu

**Keywords:** automated vehicle, model predictive controller, path following, successive linearization, vehicle dynamics

## Abstract

In this paper, a linear time-varying model predictive controller (LTV-MPC) is proposed for automated vehicle path-following applications. In the field of path following, the application of nonlinear MPCs is becoming more common; however, the major disadvantage of this algorithm is the high computational cost. During this research, the authors propose two methods to reduce the nonlinear terms: one is a novel method to define the path-following problem by transforming the path according to the actual state of the vehicle, while the other one is the application of a successive linearization technique to generate the state–space representation of the vehicle used for state prediction by the MPC. Furthermore, the dynamic effect of the steering system is examined as well by modeling the steering dynamics with a first-order lag. Using the proposed method, the necessary segment of the predefined path is transformed, the linearized model of the vehicle is calculated, and the optimal steering control vector is calculated for a finite horizon at every timestep. The longitudinal dynamics of the vehicle are controlled separately from the lateral dynamics by a PI cruise controller. The performance of the controller is evaluated and the effect of the steering model is examined as well.

## 1. Introduction

Collision avoidance systems, advanced driver-assistance systems, and the many other types of automated vehicle functions are becoming more and more popular and have become the most related topics in the field of automotive research. The advantages of automatization of different vehicle functions include the potential to improve road safety, reduce pollutant emissions and traveling times, and eliminate human errors, which are the primary cause of accidents. An automated vehicle can avoid collisions by using the steering and the braking system conventionally; furthermore, a new alternative solution for accident prevention is to force the vehicle into an unstable state, in which a control software can drive the vehicle at a level as high as a professional driver, for example, in case of a drift maneuver [1,2,3]. Each of these studies aims to drive a vehicle at the level of a professional human driver. In [1], an LQ controller is used for steady-state drifting, while in [2], an MPC is applied for drifting in varying road surface conditions, and in [3], the drift is realized by a reinforcement learning algorithm.

According to the structure of the automated vehicles, the motion planning and motion control modules are essential to achieve these goals. In this paper, the authors focus on the motion control layer by proposing an LTV-MPC structure for path-following tasks. Based on the existing research and the experimental tests, the authors identify three features to be considered in a path-following controller at higher vehicle speed, the inclusion of the vehicle dynamics in the control law, and the knowledge of the path ahead of the vehicle. Furthermore, the inclusion of the steering dynamics in the control law is identified as the third required feature, which has a high effect on the performance of the path-following controller. Numerous path-following solutions are summarized in [4,5,6], e.g., geometric controllers, such as Pure Pursuit and Stanley, linear quadratic regulators, model predictive controllers, neural network based controllers, etc. The authors decided to apply MPC for the path-following tasks, as the identified features can be incorporated into an MPC and the MPC handles constraints well. 

Three main types of MPC methods are used in the field of path tracking, the linear parameter-varying MPC (LPV-MPC) [7,8,9] the linear time-varying MPC (LTV-MPC) [10,11,12,13], and the nonlinear MPC (NLMPC) [10,14,15,16] solutions. The LPV-MPC applies a linear vehicle model for state prediction and the structure of that model does not change over time; however, a few of the variables, e.g., the velocity of the vehicle, can change. The model is calculated at each timestep based on the current states. The main advantage of the LPV-MPC method is the low computational cost; however, this technique reaches the limit of applicability when the controlled system leaves the linear region, where the system model is defined. Thus, the LPV-MPC method is unable to handle nonlinear system dynamics in a wide range, e.g., tire dynamics in the nonlinear region of the tires. The change in given parameters can be determined by measurements or estimated by state estimators.

The LTV-MPC [10,11,12,17] can handle the nonlinear behavior of the system by linearizing the nonlinear system at every timestep and estimating the states accordingly. The predicted states are still based on a linear system model; however, these are extremely close to the real states of the system, since the controller calculates the predicted states for a short horizon, e.g., a few hundred ms, in which range the prediction is acceptable. In the LTV-MPC structure, the current vehicle state is the basis of the linearization and an additional transformation in the system matrices and the control input is required. The LTV-MPC method claims a bit more computational cost than the LPV-MPC; however, it still can run on an ECU or a rapid prototyping unit in real-time. When using LPV-MPC or LTV-MPC, usually, a quadratic cost function is formulated, the optimum of which is computationally cheaper to find.

Applying the NLMPC, the evolution matrices are calculated based on a nonlinear system model without linearization. A nonlinear cost function is formulated, the optimum of which is computationally expensive to find, and might limit the applicability of the NLMPC, as discussed in [6].

In this paper, the authors propose an LTV-MPC method for automated vehicle path-following scenarios. The aim of this research is to apply a nonlinear vehicle model for state prediction, handled by a successive linearization technique, where the vehicle model is linearized at every timestep by Jacobi linearization. The applied vehicle model is coupled with a Pacejka tire model, the parameters of which are identified based on measurement results. The application of a nonlinear vehicle model coupled with the Pacejka model is expected to be suitable for handling the high sideslip angles of the tires, where the tires are operating in the nonlinear region–which the LPV-MPC is unable to handle–hence, the vehicle should be able to perform the path-following task at the limit of handling.

In the existing research, if the path is defined in a forward-looking way, it is performed by defining the reference path in the global coordinate system by a series of (*x*,*y*) points [10,12,18,19,20,21]. While this solution is obvious, it incorporates high nonlinearity into the plant model, since the motion of the vehicle needs to be transformed from the vehicle’s coordinate system–i.e., the ego frame–to the global frame. A novel method for the definition of the reference path is presented, by which the number of the nonlinear terms involved in the entire control problem is successfully reduced. According to our proposal, the path is transformed from the global coordinate system into the vehicle’s coordinate system and the reference states, which need to be realized by the vehicle, are expressed according to the state–space representation of the vehicle plant model used for state prediction by the MPC. In the existing research, the reference path is either not defined in a forward-looking way [7,22], or the forward-looking property is limited to the lateral displacement of the vehicle [8,12]. In this article, the authors define both the lateral displacement and the heading angle as a reference series for a finite horizon, as presented in Section 3. 

Some studies considered the effect of the actuator fault [23] and the robustness of the controlled system [24]; in this article, the effect of the steering system is analyzed. The effect of the consideration of the steering dynamics in the vehicle model applied for state prediction is discussed in this paper as well. The steering system is modeled as a first-order lag. Moreover, the performance of the controller is evaluated with and without the inclusion of the steering dynamics in the plant model by applying the control input to the same vehicle model, which includes the steering dynamics.

The contribution of this paper is twofold, (1) The proposed LTV-MPC method for path following includes a novel reference definition method that effectively reduces the nonlinear terms of the path-following problem by transforming the path from the global frame to the ego frame. By this transformation, the nonlinearity that needs to be managed during the Jacobian linearization is significantly reduced as the state vector has two elements less, since the position of the vehicle in the global coordinate system is not considered in the plant model. This approach removes two equations from the model while retaining the lookahead principle of the reference definition. (2) The analysis of the effect of the steering dynamics on the path-following problem are incorporated in an LTV-MPC.

The structure of this paper is as follows. Section 2 presents the vehicle model applied in the MPC for state prediction, regarding the steering dynamics and the vehicle model used for testing the controller. In Section 3, the generation of the reference path is presented. The structure of the proposed MPC and the derivation of the quadratic programming (QP) cost function is presented in Section 4. Section 5 describes the results, while Section 6 provides an analysis, in which the controller is tested on a sine wave path and the effect of the application of linear and nonlinear vehicle models in the state prediction is compared. Finally, Section 7 discusses the concluding remarks and suggestions for further research directions.

## 2. Vehicle Modelling

In this section, the applied tire and vehicle models are presented. The authors use different vehicle models for state prediction and for simulation testing. Furthermore, a Pacejka tire model is applied in both models, the parameters of which are identified from measurement data.

### 2.1. Vehicle Model for Testing

A four-wheel vehicle model is used for testing the path-following controller in a simulation environment. The roll and pitch dynamics are neglected in the vehicle model since they have no significant effect on the path-following problem. The model describes the planar dynamics of the vehicle: the angular acceleration around the vertical axis (1), the longitudinal (2), and the lateral acceleration (3).

(1) $$\dot{r}=\frac{1}{I_{z}}M_{z}$$

(2) $$a_{x}=\frac{1}{m}F_{x}^{V}+V_{y}\dot{r}$$

(3) $$a_{y}=\frac{1}{m}F_{y}^{V}-V_{x}\dot{r}\;$$

where *r* is the yaw rate, *I_z_* is the moment of inertia of the vehicle around the axis *z*, *M_z_* is the resultant torque, which rotates the vehicle around axis *z*, *a_x_* is the longitudinal acceleration, *m* is the mass of the vehicle, *F_x_^V^* is the resultant of the longitudinal forces, acting on the vehicle, *V_y_* is the lateral velocity of the vehicle in the ego coordinate system, *a_y_* is the lateral acceleration, *F_y_^V^* is the resultant of the lateral forces acting on the vehicle, and *V_x_* is the longitudinal velocity in the ego frame.

The resultant torque and forces are calculated by (4), (5), and (6)

(4) $$M_{z}=-b(F_{yRL}+F_{yRR})+s_{f}(-F_{xFL}\sin\left(\delta_{FL}\right)+F_{xFR}\sin\left(\delta_{FR}\right))+s_{r}\left(-F_{xRL}+F_{xRR}\right)+a\left(F_{yFL}\cos\left(\delta_{FL}\right)+F_{yFR}\cos\left(\delta_{FR}\right)\right)$$

(5) $$F_{x}^{V}=F_{xRL}+F_{xRR}+F_{yFL}\sin\left(\delta_{FL}\right)+F_{yFR}\sin\left(\delta_{FR}\right)+F_{xFL}\cos\left(\delta_{FL}\right)+F_{xFR}\cos\left(\delta_{FR}\right)$$

(6) $$F_{y}^{V}=F_{yRL}+F_{yRR}+F_{xFL}\sin\left(\delta_{FL}\right)+F_{xFR}\sin\left(\delta_{FR}\right)+F_{yFL}\cos\left(\delta_{FL}\right)+F_{yFR}\cos\left(\delta_{FR}\right)$$

where *a* and *b* are the distances between the center of gravity (C.G.) and the front and the rear axle, respectively, *s_f_* and *s_r_* are the front and rear track of the vehicle, respectively, *δ_FL_* and *δ_FR_* are the front-left and front-right steering angles of the individual wheels, *F_xij_* and *F_yij_* are the longitudinal and lateral forces at the individual wheels, where *i* marks the front and the rear axles, hence, *i* = *R* as rear or *i* = *F* as front, and *j* marks the left and the right wheels, hence, *j* = *L* as left or *j* = *R* as right. In this model, the authors consider a rear-wheel drive vehicle, with equally distributed traction force on the rear wheels, hence, *F_xRL_* and *F_xRR_* are equal. Furthermore, the authors assume a front-wheel steering vehicle; hence, the steering angle is solely interpreted at the front wheels.

**Figure 1:**
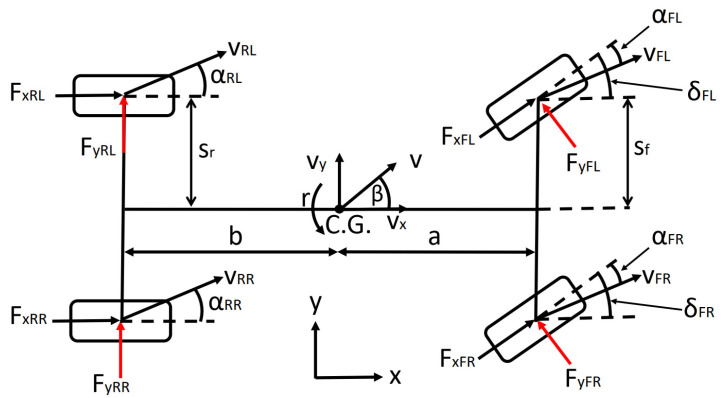
The four-wheel vehicle model applied for controller testing.

In Figure 1, the tire sideslip angles and the sideslip angle of the vehicle at C.G. are also shown. The vehicle sideslip angle is calculated by (7).

(7) $$\beta =\tan^{-1}\left(\frac{V_{y}}{V_{x}}\right)$$

The sideslip angle of an individual wheel is calculated using the velocity vector of the given wheel and the steering angle at the front axle. The velocity vector of the wheels is described by (8), the position vector of the wheels is defined by (9), where the origin is the C.G. of the vehicle, and the tire sideslip angles are described by (10) and (11).

(8) $$v_{i}=\left[\begin{array}{c} v_{xi}\\ v_{yi}\\ v_{zi} \end{array}\right]=\left[\begin{array}{c} V_{x}\\ V_{y}\\ V_{z} \end{array}\right]+\left[\begin{array}{c} 0\\ 0\\ r \end{array}\right]\times P_{i}$$

(9) $$P_{FL}={\left[\begin{array}{ccc} a & s_{f} & 0 \end{array}\right]}^{T},\;P_{FR}={\left[\begin{array}{ccc} a & -s_{f} & 0 \end{array}\right]}^{T},\;P_{RL}={\left[\begin{array}{ccc} -b & s_{r} & 0 \end{array}\right]}^{T},\;P_{RR}={\left[\begin{array}{ccc} -b & -s_{r} & 0 \end{array}\right]}^{T}\;$$

(10) $$\alpha_{FL}=\tan^{-1}\left(\frac{v_{yFL}}{v_{xFL}}\right)-\delta_{FL},\;\;\alpha_{FR}=\tan^{-1}\left(\frac{v_{yFR}}{v_{xFR}}\right)-\delta_{FR}$$

(11) $$\alpha_{RL}=\tan^{-1}\left(\frac{v_{yRL}}{v_{xRL}}\right),\;\;\alpha_{RR}=\tan^{-1}\left(\frac{v_{yRR}}{v_{xRR}}\right)$$

where *i* = *FL ∨ FR ∨ RL ∨ RR* and α denotes the tire sideslip angle. The horizontal velocity *V_z_* in (8) is considered zero as the model focuses solely on the planar dynamics of the vehicle, as stated previously.

A Pacejka tire model [20] is applied to calculate the lateral forces at each wheel. During this research, the vehicle moves at constant speed; thus, the longitudinal forces are small, especially compared to the lateral forces and, hence, solely a lateral tire model is used in this article. The Pacejka tire model is described by Equations (12) and (13) [25].

(12) $$Y=D\sin\left(C\tan^{-1}\left(B\phi \right)\right)+S_{v}$$

(13) $$\phi =\left(1-E\right)\left(\alpha +S_{h}\right)+\left(E/B\right)\tan^{-1}\left(B\left(\alpha +S_{h}\right)\right)$$

where *B* is the stiffness factor, *C* is the shape factor, *D* is the peak factor, *E* is the curvature factor, *S_v_* and *S_h_* are the vertical and the horizontal shift, respectively, and the α sideslip angle is defined in degrees. The Pacejka tire model and the meaning of the factor variables are described in more detail in [25]. 

The entire vehicle model, including the tire model, is fitted to a test vehicle used for automated vehicle function tests, such as automated drift, Moose test, and other path-following tasks. Further details of the test vehicle setup can be found in [1].

**Figure 2:**
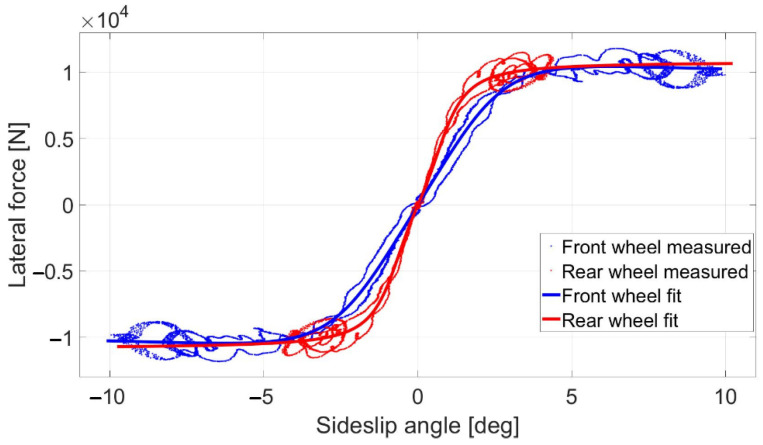
The measured and identified tire characteristics.

To determine the characteristics of the tires, a ramp steer maneuver is taken by the test vehicle and the necessary variables are measured with the data acquisition system. The parameters in the Pacejka tire model are fitted to the measurement results, as shown in Figure 2.

The characteristics of the rear tires have a greater slope at small sideslip angles, where the tire model is nearly linear. That means the rear tires have greater cornering stiffness than the front tires. This meets the expectations of the authors, since the front wheels of the vehicle are mounted with 245/35 R19 tires, while 265/35 R19 tires are applied at the rear wheels—using the same sidewall height, the wider tire becomes stiffer. During the identification of the tire characteristics, the vertical load of the tires is assumed to be constant.

The identified tire models describe the characteristics of the tires located on the same axle, not the individual wheels; hence, the results in Figure 2 contain the lateral forces generated by the two front tire pairs and the two rear tire pairs, respectively. Thus, when modeling one wheel, the value of the lateral force needs to be halved.

### 2.2. Vehicle Model for State Prediction

**Figure 3:**
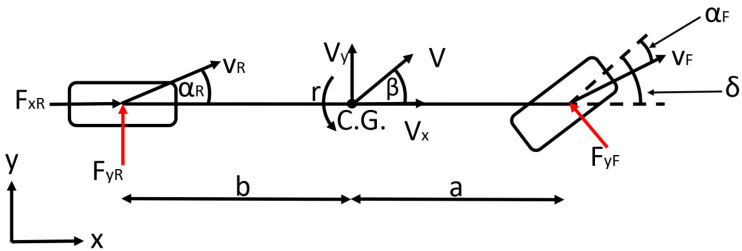
The vehicle model applied to the MPC for state prediction.

The states of the vehicle model applied in the MPC for state prediction are advisable to choose in a way to correspond to the control purpose. In this case, the aim is to drive the vehicle along a predefined path as fast as the vehicle is still able to execute the maneuver. The applied vehicle model shown in Figure 3 is a dynamic bicycle model, coupled with different Pacejka tire models at the front and the rear wheels. Since the bicycle model applies one wheel per axle, the identified Pacejka models can be used without halving the values of the lateral forces of the tire characteristics. The applied vehicle model describes the dynamics of the vehicle with four equations, enhanced by a fifth equation for the steering dynamics. The steering dynamics are considered to take into account the dynamic lag of the steering system, in which way the controller considers that the demanded steering angle will only be realized with a time delay. The dynamics of the steering system are modeled by a first-order lag, which has one tuning parameter, the time constant, to identify the measurements made. The time constant is determined by measurements conducted on the test vehicle.

The state vector of the system is chosen as *x =* [$v\;V_{y}\;\varphi \;r$ $\delta_{act}$]*^T^*, where *y* is the lateral displacement of the vehicle in the ego frame, *φ* is the heading angle of the vehicle, and *δ_act_* is the actual steering angle. The vehicle dynamics are described by (14)–(17), while the steering dynamics by (18).

(14) $$V_{y}=\dot{y}$$

(15) $$a_{y}={\dot{V}}_{y}=\left(\frac{F_{yf}\cos\left(\delta \right)+F_{yr}}{m}\right)-V_{x}r$$

(16) $$r=\dot{\varphi }$$

(17) $$\dot{r}=\frac{aF_{yf}\cos\left(\delta \right)-bF_{yr}}{I_{z}}$$

(18) $${\dot{\delta }}_{act}=-\frac{1}{T_{st}}\delta_{act}+\frac{1}{T_{st}}\delta_{dem}$$

where *δ_dem_* is the steering angle required by the MPC and *T_st_* is the time constant of the first-order steering model. To calculate the *F_yf_* and *F_yr_* lateral forces, the identified Pacejka models are applied by determining the parameters of (12) and (13). The sideslip angles are calculated by (19) and (20).

(19) $$v_{yF}={\dot{v}}_{y}+ar,\;v_{yR}={\dot{v}}_{y}-br$$

(20) $$\alpha_{F}=\tan^{-1}\left(\frac{v_{yF}}{v_{xF}}\right)-\delta_{F},\;\alpha_{R}=\tan^{-1}\left(\frac{v_{yR}}{v_{xR}}\right)$$

where *v_yF_* and *v_yR_* are the velocity of the front and the rear wheels and *α_F_* and *α_R_* are the sideslip angles of the front and the rear wheels, respectively. The tests are conducted without the consideration of the steering dynamics, in which case, the state vector is chosen as *x =* [$v\;V_{y}\;\varphi \;r$]*^T^*, and (18) is neglected during the state prediction.

The future states of a vehicle using a vehicle model can be predicted for a finite time horizon. In this article, the authors apply successive linearization to continuously generate the state–space representation of the vehicle. The successive linearization allows linearizing the nonlinear vehicle model at the current operating point, calculating the state–space representation accordingly, and making the state prediction based on the linearized system. The linearization is conducted at every timestep; hence, the MPC can use the latest state of the vehicle as a basis of the state prediction.

The linearization of the vehicle model is solved by Jacobian linearization and leads to the continuous-time [*A_c_*, *B_c_*, *C_c_*, *D_c_*] state–space representation of the system (21).

(21) $$A_{c}\left(i,j\right)=\frac{\partial F_{i}}{\partial x_{j}},\;B_{c}\left(i,j\right)=\frac{\partial F_{i}}{\partial u_{j}},\;C_{c}\left(i,j\right)=\frac{\partial z_{i}}{\partial x_{j}},\;D_{c}\left(i,j\right)=\frac{\partial z_{i}}{\partial u_{j}}$$

where *F* is the system of nonlinear equations (14)–(18), *x* is the state vector, *u* is the control input, which, in this case, is the demanded steering angle *δ_dem_*, while *C_c_* and *D_c_* are considered constant matrices (22).

(22) $$C_{c}=\left[\begin{array}{ccccc} 1 & 0 & 0 & 0 & 0\\ 0 & 0 & 1 & 0 & 0 \end{array}\right],\;\;D_{c}=\left[\begin{array}{c} 0\\ 0 \end{array}\right]$$

Furthermore, matrix *B_c_* remains constant during the linearization in the following form (23).

(23) $$B_{c}={\left[\begin{array}{ccccc} 0 & 0 & 0 & 0 & \frac{1}{T_{st}} \end{array}\right]}^{T}$$

The partial derivatives of the matrices are evaluated at the desired operation point, which is specified by the state vector *x_o_*, the time derivative of the state vector ${\dot{x}}_{o}$, and the control vector *u_o_* and, thereby, the partial derivatives in (21) need to be calculated based on the initial conditions $x=x_{o}$, $\dot{x}={\dot{x}}_{o}$ and $u=u_{o}$. The evolution of the continuous-time system can be described by (24)

(24) $${\dot{x}}_{L}={\dot{x}}_{o}+A_{c}\left(x_{L}-x_{o}\right)+B_{c}\left(u_{L}-u_{o}\right)$$

where *x_L_* and *u_L_* are the linearized state of the system and the control input, respectively. The constant terms of (24) are incorporated into *K_c_*, with which the continuous-time representation of the state–space system becomes (25) and (26).

(25) $${\dot{x}}_{L}=A_{c}x_{L}+B_{c}u_{L}+K_{c},\;\;K_{c}={\dot{x}}_{o}-A_{c}x_{o}-B_{c}u_{o}$$

(26) $$y_{L}=C_{c}x_{L}+D_{c}u_{L}$$

where *y_L_* is the output of the system.

Since the MPC is a discrete-time-control technique, the state–space representation needs to be discretized to get the discrete-time state–space representation of the system [*A_d_, B_d_, C_d_, D_d_*] and *K_d_* (27)–(30).

(27) $$A_{d}=e^{A_{c}T_{s}}$$

(28) $$B_{d}=A_{c}^{-1}\left(e^{A_{c}T_{s}}-I\right)B_{c}$$

(29) $$K_{d}=A_{c}^{-1}\left(e^{A_{c}T_{s}}-I\right)K_{c}$$

(30) $$C_{d}=C_{c},\;\;D_{d}=D_{c}$$

where *T_s_* is the discretization timestep, which is equal to the timestep value of the MPC. Finally, the discrete-time representation of the system (31) can be used for state prediction by the MPC.

(31) $$x\left(k+1\right)=A_{d}x\left(k\right)+B_{c}u\left(k\right)+K_{d}\;\;y\left(k+1\right)=C_{d}x\left(k+1\right)+D_{d}u\left(k+1\right)$$

## 3. Reference Path Definition

**Figure 4:**
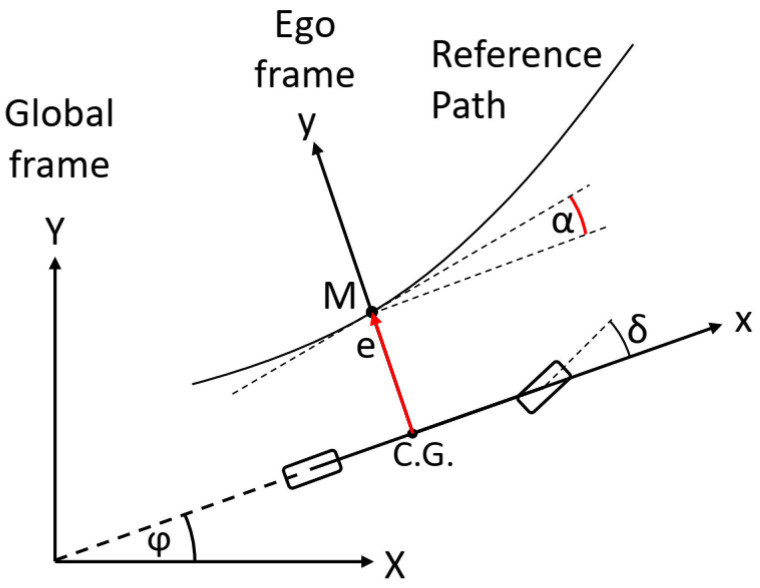
The interpretation of the lateral and angular errors.

As previously introduced, in numerous studies, the control problem is to minimize the error between the spatial reference path and the position of the vehicle. In this research, the state–space representation of the vehicle contains the position of the vehicle in the global frame, according to the reference definition, where the path is defined by a series of (*x*,*y*) points in the global frame. The controlled state is the spatial position of the vehicle—X and Y coordinates in the global frame. The spatial path-following problem is possibly supplemented with the tracking of the heading and the yaw rate. The problem with the formulation when the X and Y coordinates are defined as a reference is that it introduces several nonlinear terms into the vehicle model applied for state prediction by the MPC and increases the dimension of the state vector by two. Therefore, if the control problem is defined to follow spatial points, the vehicle needs to be transformed to the global frame in the state–space representation—this transformation is responsible for higher nonlinearity. Thus, if the increase in the dimension of state vector *x* by two—displacement in X and Y directions—can be omitted, then the computational requirements of the MPC can be reduced, which increases directly proportionally with the increase in the dimension of state vector *x*, as presented in [26]. In this article, a novel method is proposed for the reduction in nonlinear terms, which is to transform the path to the vehicle and describe the path-following task in a way that retains the advantageous property of the MPC, which is the knowledge of the path ahead of the vehicle—the knowledge of the reference for a finite horizon. In this article, the lateral displacement and the heading of the vehicle are defined as a reference to be followed, in accordance with the states of the vehicle model used for state prediction. The reference state variables are chosen as they clearly define the relationship between the vehicle and the path, with respect to the lateral terms. To calculate the reference for a finite horizon, firstly, the lateral and angular errors are defined, as shown in Figure 4. The lateral error *e* is the distance between the C.G. point of the vehicle and the intersection point *M* on the *y* axis of the ego frame and the reference path.

**Figure 5:**
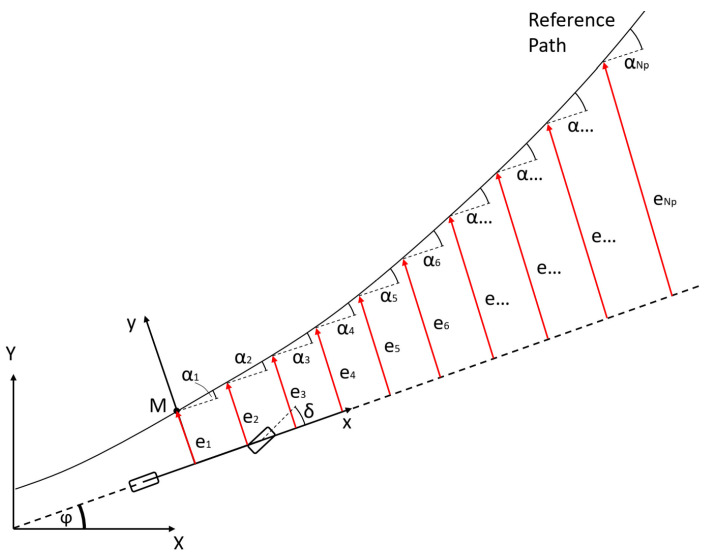
The interpretation of the lateral error reference and orientation angle reference in both ego frame and global frame.

The angular error *α* is the angle between the heading of the vehicle and the tangent of the path at the intersection point *M*. To generate the reference for the lateral displacement state *y* and for the heading angle state *φ*, the vehicle, the reference path, the ego frame, and the global frame need to be considered, as shown in Figure 5. In Figure 5, the lateral errors and the angle errors are shown for a finite *N_p_* horizon, where *N_p_* is the prediction horizon of the MPC. If the lateral error values from the ego coordinate system can be seen, these errors can be interpreted as reference lateral displacements, which corresponds to *y*, the first state of the state–space representation. In a similar way, the angular errors can be interpreted as *φ* reference heading angles. By applying this method for reference generation, the transformation of the vehicle coordinates from the ego frame to the global frame becomes avoidable, resulting in omitting several nonlinear terms from the vehicle model.

As stated before, if the vehicle model is not transformed into the global frame, the path needs to be transformed into the ego frame of the vehicle. The transformation is defined by Equations (32)–(34),

(32) $$\gamma =\alpha_{1}+\varphi$$

(33) $$H=\left[\begin{array}{c} 0\\ e_{1} \end{array}\right]$$

(34) $$P_{ref,i}=H+\left[\begin{array}{cc} \cos\left(\gamma \right) & -\sin\left(\gamma \right)\\ \sin\left(\gamma \right) & \cos\left(\gamma \right) \end{array}\right]P_{i}$$

where *γ* is the transformation angle, *e_1_* and *α_1_* are the lateral and angular errors at the *M* point, respectively, *H* is an offset vector, which shifts the entire reference trajectory to the necessary lateral displacement, *P_i_* is the (*x*,*y*) coordinate of the *i*th path point described in the global frame, and *P_ref,i_* is the coordinate of the reference path point in the ego frame. After the transformation, the lateral error references and the angular error references can be calculated; the lateral error reference is the *y*-direction component of *P_ref,i_*, which determines the series of *e_1_, e_2_, … e_Np_*, while the angular reference can be calculated considering the tangent of the transformed path at every point where the lateral displacements are defined.

After the reference values are calculated, the reference vector can be generated by (35) and (36).

(35) $$t=\left[\begin{array}{c} e_{i}\\ \alpha_{i} \end{array}\right]$$

(36) $$Τ=\left[\begin{array}{cccc} t_{1} & t_{2} & \cdots & t_{N}{}_{p} \end{array}\right]{\;}^{T}=\left[\begin{array}{ccccccc} e_{1} & \alpha_{1} & e_{2} & \alpha_{2} & \cdots & e_{N_{p}} & \alpha_{N}{}_{p} \end{array}\right]{\;}^{T}$$

While the original reference path is described by *N_p_* points, the reference lateral displacements and the heading angles need to be calculated for every path point. The result is a stacked matrix *T* with a dimension of *2*× *N_p_*. Using this method of reference definition, several nonlinear terms are omitted from the vehicle model, the reference is coherent to the states of the vehicle model, the reference lateral displacement is coupled with the first state in the state–space model, and the angle reference is coupled with the third state. Furthermore, the presented reference generation method preserves the forward-looking nature of the problem definition, which is necessary for the path-following task.

## 4. MPC Structure

As stated in Section 2, the authors apply a successive linearization technique, similar to [12,14,17], to handle the nonlinear vehicle model. During the successive linearization, the nonlinear vehicle model is linearized at every timestep based on the actual state vector of the vehicle. The resulting state–space representation is applied to predict the future vehicle states for a finite prediction horizon, the length of which is *N_p_*. Using an MPC controller, the objective is to minimize the difference between the given reference states in the system and the predicted states in the systems by calculating the optimal control input vector as a result of an optimization process, while meeting a set of constraints. The optimization process requires a cost function to minimize. In this section, the derivation of the cost function is presented. The cost function depends on the tracking error and the amount of the control input. The tracking error *e*(*k*) in the *k*-th timestep is defined as *e*(*k*) = *y*(*k*) − *r*(*k*), where *y*(*k*) is the current state of the system and *r*(*k*) is the given reference. Then, the evolution of the error can be defined for the *k*-th, *k* + 1-th, and *k* + 2-th timesteps, etc., by (37).

(37) $$e\left(k\right)=C_{d}x\left(k\right)+D_{d}u\left(k\right)-r\left(k\right)\;e\left(k+1\right)=C_{d}x\left(k+1\right)+D_{d}u\left(k+1\right)-r\left(k+1\right)\;=C_{d}A_{d}x\left(k\right)+C_{d}B_{d}u\left(k\right)+C_{d}K_{d}+D_{d}u\left(k+1\right)-r\left(k+1\right)\;e\left(k+2\right)=C_{d}x\left(k+2\right)+D_{d}u\left(k+2\right)-r\left(k+2\right)=\;C_{d}A_{d}^{2}x\left(k\right)+C_{d}A_{d}B_{d}u\left(k\right)+C_{d}B_{d}u_{d}\left(k+1\right)+C_{d}A_{d}K_{d}+C_{d}K_{d}+\;D_{d}u\left(k+2\right)-r\left(k+2\right)$$

The evolution of the error needs to be calculated for the entire prediction horizon, resulting in the error vector $\bar{e}\in R^{N_{p}N_{o}}$, where *N_o_* is the number of outputs in the system, which is equal to the rows of matrix *C_d_*. In this case, *N_o_* = 2 corresponds to the lateral displacement and the heading angle references.

(38) $$\bar{e}=\overset{=}{P}x\left(k\right)+\overset{=}{H}\bar{u}+\overset{=}{E}K_{d}-\bar{r}$$

(39a) $$\bar{e}=\left[\begin{array}{c} e\left(k\right)\\ \begin{array}{c} e\left(k+1\right) \end{array}\\ e\left(k+2\right)\\ \vdots \\ e\left(k+N_{p}-1\right) \end{array}\right],\;\;\overset{=}{P}=\left[\begin{array}{c} C_{d}\\ C_{d}A_{d}\\ \begin{array}{c} C_{d}A_{d}^{2} \end{array}\\ \vdots \\ C_{d}A_{d}^{N_{p}-1} \end{array}\right]$$

(39b) $$\overset{=}{H}=\left[\begin{array}{cccc} D_{d} & 0 & 0 & \ldots \\ C_{d}B_{d} & D_{d} & 0 & \ldots \\ C_{d}A_{d}B_{d} & C_{d}B_{d} & D_{d} & \ldots \\ \vdots & \vdots & \vdots & \ddots \\ C_{d}A_{d}^{N_{p}-2}B_{d} & C_{d}A_{d}^{N_{p}-3}B_{d} & C_{d}A_{d}^{N_{p}-4}B_{d} & \ldots \end{array}\right]$$

(39c) $$\bar{u}=\left[\begin{array}{c} u\left(k\right)\\ u\left(k+1\right)\\ u\left(k+2\right)\\ \vdots \\ u\left(k+N_{p}-1\right) \end{array}\right],\;\;\overset{=}{E}=\left[\begin{array}{c} 0\\ C_{d}\\ C_{d}\left(I+A_{d}\right)\\ \vdots \\ C_{d}\left(I+\sum_{i=1}^{N_{p}-2}A_{d}^{i}\right) \end{array}\right]$$

(39d) $$\bar{r}=\left[\begin{array}{c} r\left(k\right)\\ \begin{array}{c} r\left(k+1\right) \end{array}\\ r\left(k+2\right)\\ \vdots \\ r\left(N_{p}-1\right) \end{array}\right]$$

where $\bar{u}\in R^{N_{p}N_{u}}$ is the control input vector, i.e., the result of the optimization process, *N_u_* is the number of the control inputs, which is equal to the columns of matrix *B_d_*—in this case—*N_u_* = 1 corresponding to the steering input, $\overset{=}{P}\in R^{N_{p}N_{o}\times N_{x}}$, *N_x_* is the dimension of the state vector, $\overset{=}{H}\in R^{N_{p}N_{o}\times N_{p}N_{u}}$, $\overset{=}{E}\in R^{N_{p}N_{o}\times N_{x}}$, $\bar{r}\in R^{N_{p}N_{o}}$ is the reference matrix, which defines the reference for the *N_p_* horizon. In (39), *P*, *H*, and *E* are the error evolution matrices. The constant terms in (38) can be combined as $\overset{=}{K}=\overset{=}{E}K_{d}-\bar{r}$, which leads to a more compact form of $\bar{e}$.

(40) $$\bar{e}=\overset{=}{P}x\left(k\right)+\overset{=}{H}\bar{u}+\overset{=}{K}$$

As the evolution of the tracking error is calculated, the cost function can be defined as

(41) $$J\left(k\right)=\frac{1}{2}\left(\bar{e}{\left(k\right)}^{T}\overset{=}{Q}\bar{e}\left(k\right)+\bar{u}{\left(k\right)}^{T}\overset{=}{R}\bar{u}\left(k\right)\right)$$

where $\overset{=}{Q}\in R^{N_{p}N_{o}\times N_{p}N_{o}}$ penalizes the deviation from the reference states and $\overset{=}{R}\in R^{N_{p}N_{u}\times N_{p}N_{u}}$ penalizes the number of the control input. Both $\overset{=}{Q}$ and $\overset{=}{R}$ are diagonal square matrices, built by using matrix $\overset{=}{q}$ and scalar r, according to (42) and (43), where *q_1_* and *q_2_* are the weights of the deviation from the reference lateral distance and the heading angle, respectively, and *r* is the weight of the control input.

(42) $$\overset{=}{Q}=\left[\begin{array}{cccc} \overset{=}{q} & 0 & \ldots & 0\\ 0 & \overset{=}{q} & \ldots & 0\\ \vdots & \vdots & \ddots & 0\\ 0 & 0 & \ldots & \overset{=}{q} \end{array}\right],\;\;\overset{=}{R}=\left[\begin{array}{cccc} r & 0 & \ldots & 0\\ 0 & r & \ldots & 0\\ \vdots & \vdots & \ddots & 0\\ 0 & 0 & \ldots & r \end{array}\right]$$

(43) $$\overset{=}{q}=\left[\begin{array}{cc} q_{1} & 0\\ 0 & q_{2} \end{array}\right],\;\;r=\left[r\right]$$

In this article, the authors apply a quadratic cost function (41), the optimum of which is found by the built-in numerical solver in the MATLAB software package, called *quadprog*. Substituting (40) into (41) results in

(44) $$J\left(k\right)=\frac{1}{2}\left({\left(\overset{=}{P}x\left(k\right)+\overset{=}{H}\bar{u}+\overset{=}{K}\right)}^{T}\overset{=}{Q}\left(\overset{=}{P}x\left(k\right)+\overset{=}{H}\bar{u}+\overset{=}{K}\right)+\bar{u}{\left(k\right)}^{T}\overset{=}{R}\bar{u}\left(k\right))\right)$$

The following task is to aggregate the terms that do not depend on the control input $\bar{u}$ and aggregate the quadratic and linear terms of (44), which leads to (45).

(45) $$J\left(k\right)=\frac{1}{2}\bar{u}{\left(k\right)}^{T}\overset{\overset{=}{G}}{\overbrace{\left(\overset{=}{H^{T}}\overset{=}{Q}\overset{=}{H}+\overset{=}{R}\right)}}\bar{u}\left(k\right)+\overset{\overset{=}{W^{T}}}{\overbrace{\left(x{\left(k\right)}^{T}\overset{=}{P^{T}}+\overset{=}{K^{T}}\right)\overset{=}{Q}\overset{=}{H}}}\bar{u}\left(k\right)$$

A more compact form of the cost function is in (46).

(46) $$J\left(k\right)=\frac{1}{2}\bar{u}{\left(k\right)}^{T}\overset{=}{G}\bar{u}\left(k\right)+\overset{=}{W^{T}}\bar{u}\left(k\right)$$

When solving optimal control problems, the aim is to minimize the tracking errors and the amount of control input. However, a steady-state error might occur while tracking, since the cost of the steady-state error might be less than the cost of the higher value of the control inputs. To avoid the steady-state error, the cost function is defined by the increments in the current control input, rather than defining the individual control inputs. Applying this solution, the effect of the control input values on the cost function is minimal. The control input vector can be transformed as

(47) $$u\left(k\right)=u\left(k-1\right)+\rho u_{1}\;u\left(k+1\right)=u\left(k\right)+\rho u_{2}=u\left(k-1\right)+\rho u_{1}+\rho u_{2}\;u\left(k+N_{p}-1\right)=u\left(k+N_{p}-2\right)+\rho u_{N_{p}}=u\left(k-1\right)+\rho u_{1}+\rho u_{2}+\ldots +\rho u_{N_{p}}\;$$

which can be written as

(48) $$\overset{\bar{u}}{\overbrace{\left[\begin{array}{c} u\left(k\right)\\ \begin{array}{c} u\left(k+1\right) \end{array}\\ \vdots \\ u\left(k+N_{p}-1\right) \end{array}\right]}}=\overset{\bar{u}\left(k-1\right)}{\overbrace{\left[\begin{array}{c} u\left(k-1\right)\\ u\left(k-1\right)\\ \vdots \\ u\left(k-1\right) \end{array}\right]}}+\overset{\overset{=}{D}}{\overbrace{\left[\begin{array}{ccc} I & 0 & \ldots \\ I & I & \ldots \\ \vdots & \vdots & \ddots \\ I & I & \ldots \end{array}\right]}}\overset{\rho \bar{u}\left(k\right)}{\overbrace{\left[\begin{array}{c} \rho u_{1}\\ \rho u_{2}\\ \vdots \\ \rho u_{N_{p}} \end{array}\right]}}$$

where $\overset{=}{D}\in R^{N_{p}N_{u}\times N_{p}N_{u}}$ is a lower diagonal matrix built of $I\in R^{N_{u}\times N_{u}}$ matrices and zero matrices of the same dimensions. Performing the transformation, the evolution of the tracking error can be written as

(49) $$\bar{e}=\overset{=}{P}x\left(k\right)+\overset{=}{H}\left(\bar{u}\left(k-1\right)+\overset{=}{D}\rho \bar{u}\left(k\right)\right)+\overset{=}{K}$$

where $\bar{u}\left(k-1\right)$ is constant for the entire prediction horizon, thus, it can be included in the constant term $\overset{=}{K}$. The new const function, the solution of which is the optimal series of control input increments, is given in the following form

(50) $$J_{D}\left(k\right)=\frac{1}{2}\rho \bar{u}{\left(k\right)}^{T}{\overset{=}{G}}_{D}\rho \bar{u}\left(k\right)+\overset{=}{W_{D}^{T}}\rho \bar{u}\left(k\right)$$

where the matrices are defined as ${\overset{=}{G}}_{D}=D^{T}\bar{\overset{=}{G}}D$ and $\overset{=}{W_{D}^{T}}={\bar{u}}^{T}\left(k-1\right)\bar{\overset{=}{G}}D$.

## 5. Results

**Figure 6:**
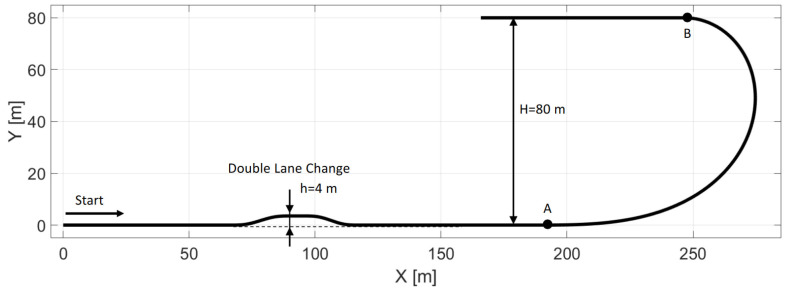
The reference path.

A reference path is defined for testing the controller shown in Figure 6. The path contains a double-lane-change section, which is considered an evasive maneuver [27], and a U-turn section, which is built by a clothoid segment, marked with *A* and *B* in Figure 6. As the vehicle drives along the U-turn section, the curvature of the path is the linear function of the length of the arc. During the simulations, the vehicle is started from the left side of the path, using a 0.2 m offset, which is applied to examine how the vehicle can find the path. The simulations are conducted at 50, 60, and 70 km/h, using vehicle models, excluding and including the steering dynamics; hence, in total, six cases are examined. During the simulations, the identified model of the steering system is applied to the vehicle model in every case; hence, the consideration of the steering dynamics in the prediction model is expected to lead to better results. The value of *N_p_* and *N_c_* is 10 and the sampling time is 0.05 s.

The results are summarized in Table 1, where *e* is the lateral error and *ϕ* is the orientation error. As the velocity of the vehicle is increased, the average and maximal errors are also increased; furthermore, the application of the steering dynamics can reduce the errors significantly.

**Figure 7:**
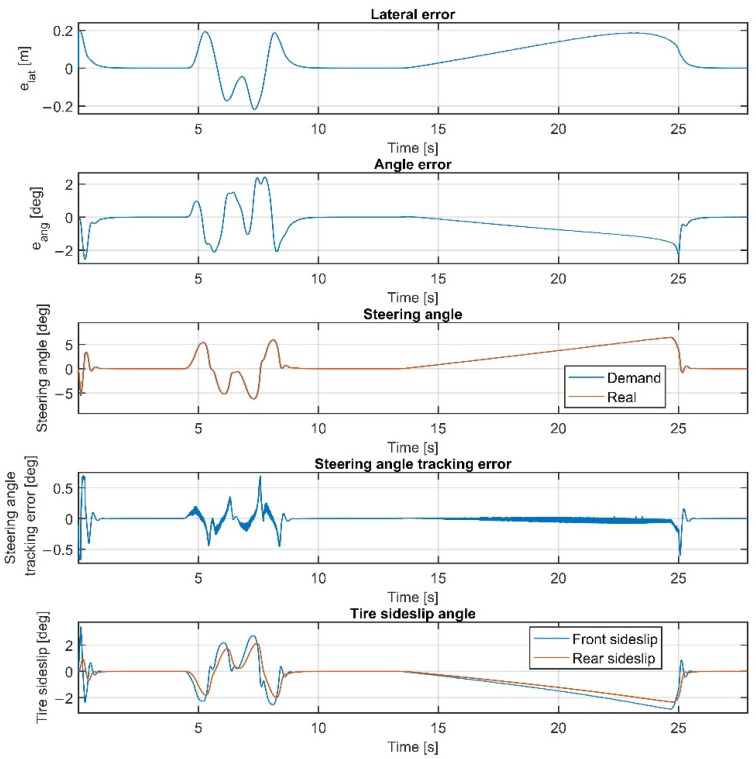
Simulation results at 50 km/h, without steering dynamics.

**Figure 8:**
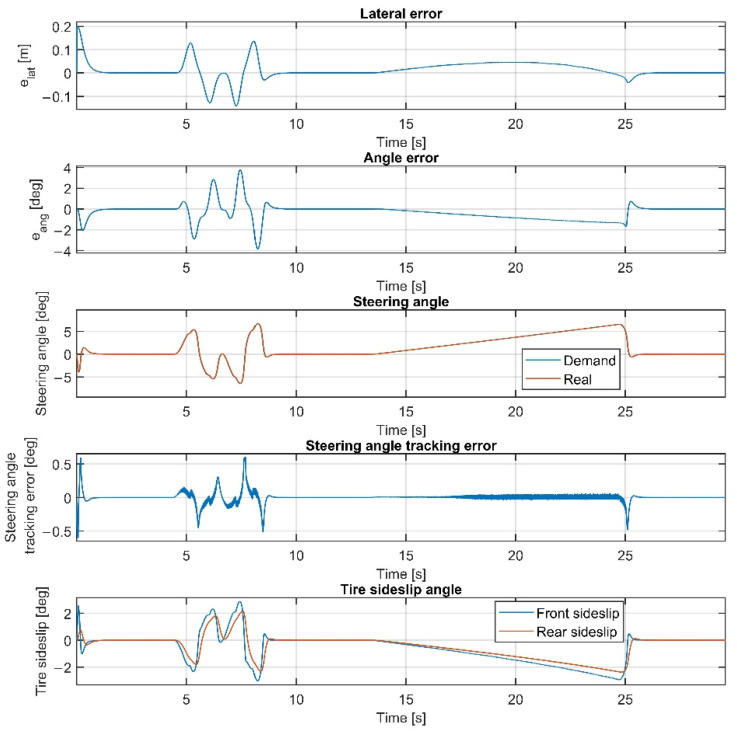
Simulation results at 50 km/h, including steering dynamics.

Please note that the figures below sometimes do not show the difference between the reference and the real steering angle, which is due to the accurate reference tracking. As shown in Figure 7 and Figure 8, at 50 km/h, the consideration of the steering dynamics can increase the accuracy of the controller and result in a smoother steering command, which increases the stability of the vehicle and provides better ride comfort to the passengers. Furthermore, smaller steering interventions result in smaller sideslip angles.

**Figure 9:**
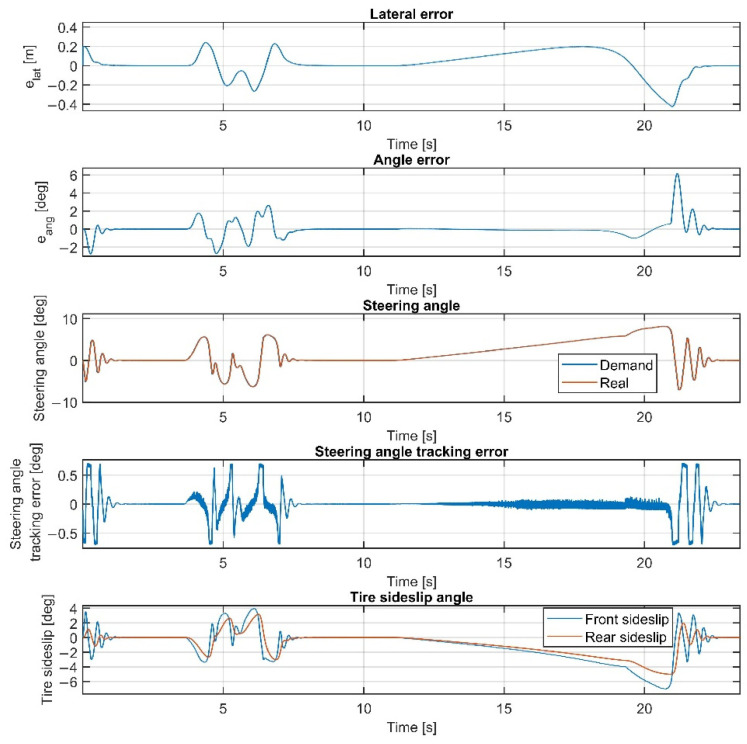
Simulation results at 60 km/h, without steering dynamics.

**Figure 10:**
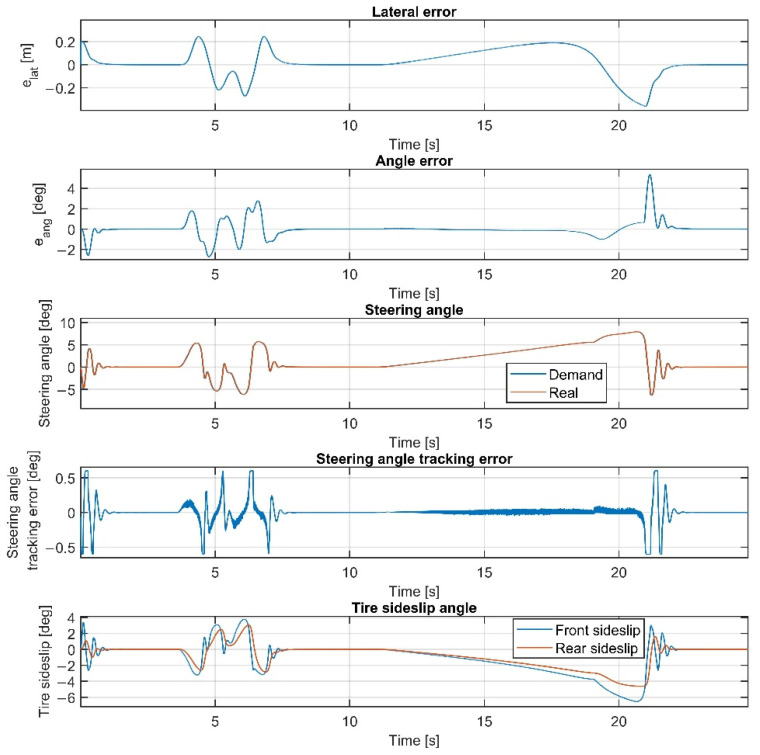
Simulation results at 60 km/h, including steering dynamics.

The result at 60 km/h is shown in Figure 9 and Figure 10. The results show similar behavior to the 50 km/h cases; however, the errors are a little higher, although still in an acceptable range. As shown in Figure 2, the tires become saturated when reaching about 4 deg and 2 deg of sideslip at the front and the rear wheels, respectively.

The tire sideslips reach about 7 deg at the front wheels and 5 deg at the rear wheels, which means the tires operate in a saturated state when the vehicle drives at the ending section of the U turn, resulting in a higher lateral error there.

**Figure 11:**
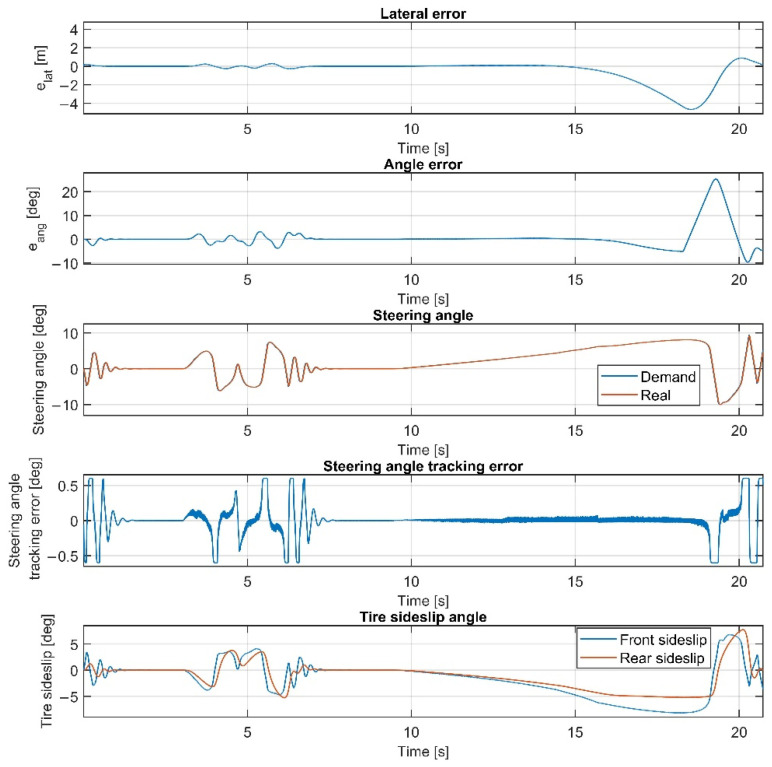
Simulation results at 70 km/h, without steering dynamics.

**Figure 12:**
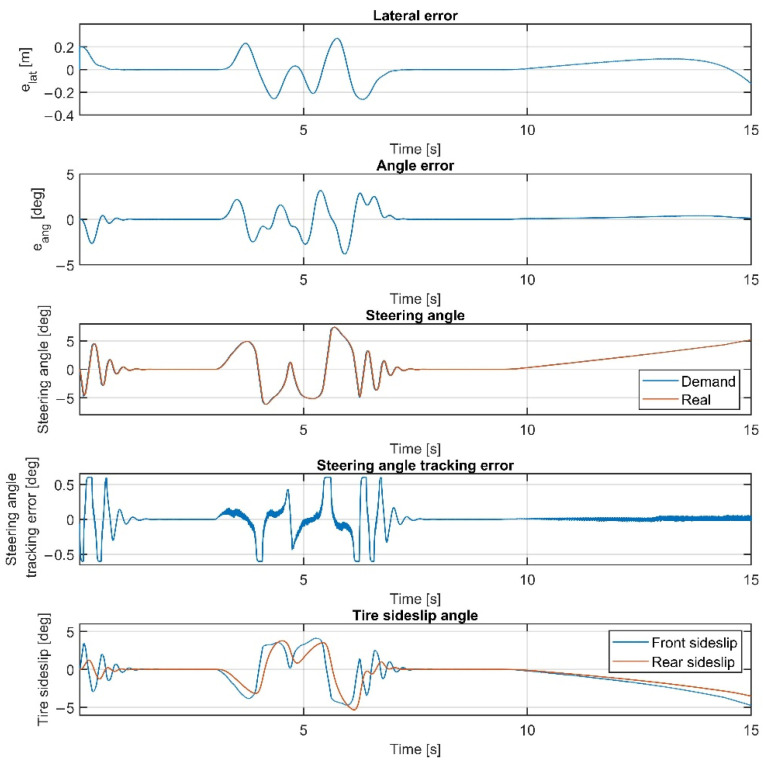
Simulation results at 70 km/h, without steering dynamics in 0–15 sec interval.

**Figure 13:**
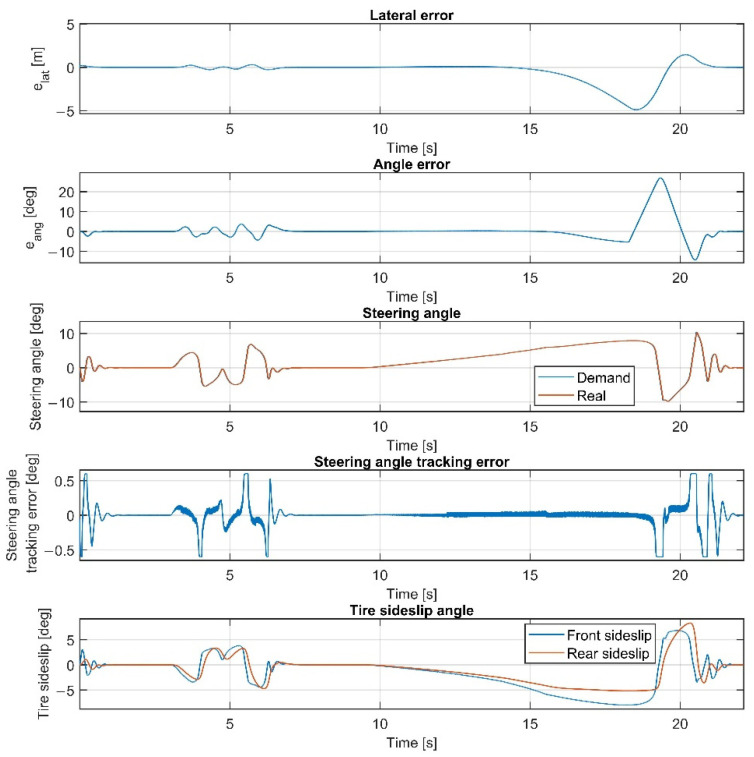
Simulation results at 70 km/h, including steering dynamics.

**Figure 14:**
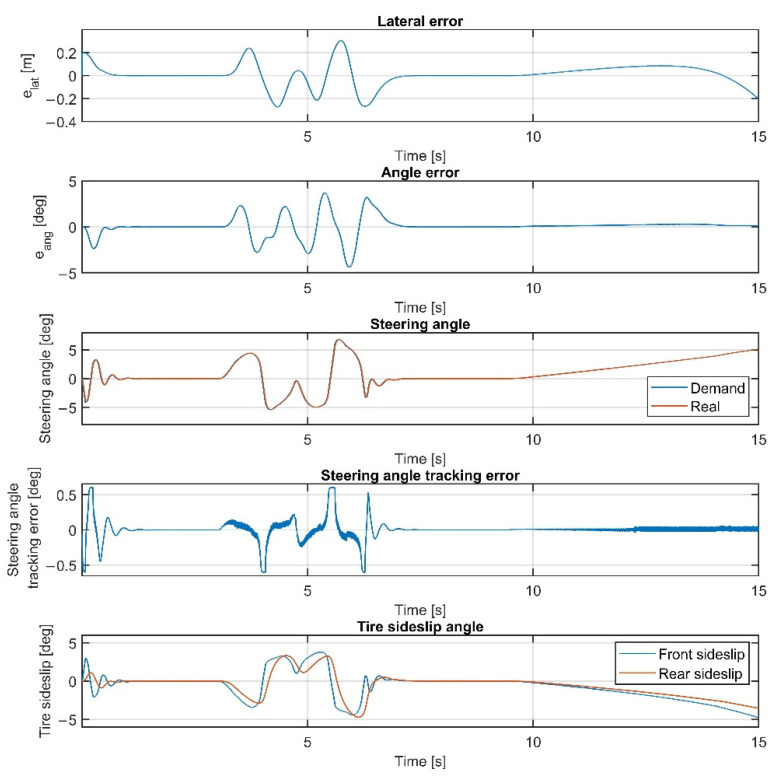
Simulation results at 70 km/h, including steering dynamics in 0–15 sec interval.

The results at 70 km/h are shown in Figure 11, Figure 12, Figure 13 and Figure 14. Figure 14 and Figure 12 show the same results as Figure 13 and Figure 11, respectively, but in a smaller time window to make the results more visible. The vehicle reaches and exceeds the friction limit at the U turn, which leads to high lateral errors—the vehicle is physically not able to follow the path due to its great curvature.

The importance of the 70 km/h case is to show whether the controller can handle the vehicle at the friction limit or increase the steering angle to a high value, applying unnecessarily large sideslip angles, which can not decrease the path-following errors. According to the simulation results, the controller can handle the nonlinear characteristics of the tires and operates stably, even when the sideslip angles reach high values. As shown in Figure 11 and Figure 13, the sideslip angles reach a maximal 8 deg and 5 deg at the front and the rear wheels, respectively. The further increase in the sideslip would not generate a larger lateral force. As the cost function penalizes the value of the steering intervention, the steering angle does not increase. In this research, the authors do not apply constraints on the sideslip angle; however, the controller does not generate too-large values due to the inclusion of the nonlinear Pacejka tire model in the plant model.

In the double-lane-change section, the errors do not show a significant increase compared to the lower-speed scenarios; the controller could drive the vehicle faster in this section, but the higher speed would result in a greater error at the U-turn. Please consider that in Table 1, the high values of the average errors at 70 km/h are caused by the large errors at the U-turn, which has a serious effect when calculating the average errors.

Overall, the inclusion of the steering dynamics in the plant model leads to a more accurate path following, with a smoother steering intervention, while the complexity of the model is not seriously increased. Furthermore, as shown by the fourth subgraphs in Figure 7, Figure 8, Figure 9, Figure 10, Figure 11, Figure 12, Figure 13 and Figure 14, the inclusion of the steering dynamics in the plant model results in a more accurate steering demand tracking, where the tracking error is decreased in every case.

## 6. Evaluation of the Effectiveness of the Controller and Comparison of the Linear/Nonlinear Plant Model

**Figure 15:**
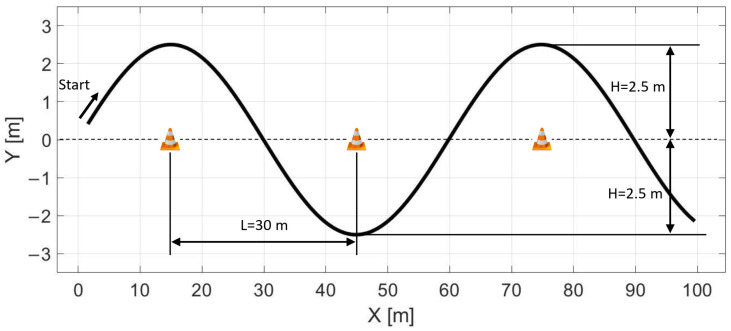
Sine wave reference path.

In this section, the effectiveness of the controller is evaluated by testing on a sine wave path. Furthermore, the effect of the vehicle plant model is also evaluated by comparing the results of a linear vehicle model and a nonlinear vehicle model. The reference path is shown in Figure 15. The distance between the cones is 30 m, which means 60 m wavelength, and the lateral peak value of the sine wave is 2.5 m in both directions. Each test scenario presented in this section is conducted using the four-wheel vehicle model presented in Section 2.1 and the plant model contains the steering dynamics in each test case.

**Figure 16:**
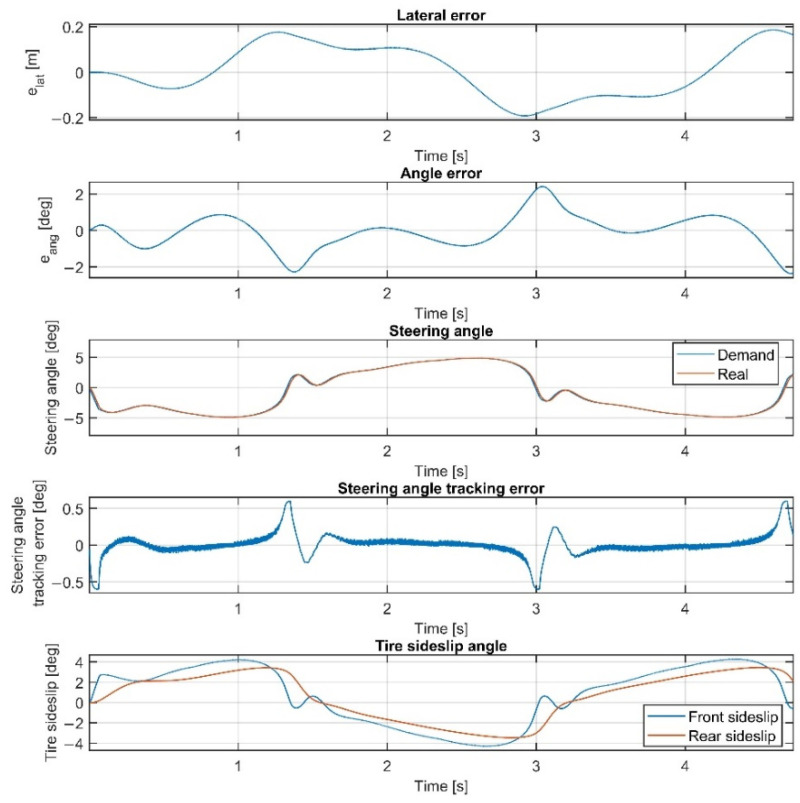
Simulation results on the sine wave path at 70 km/h, including steering dynamics, using the nonlinear vehicle model for state prediction.

The results of the scenarios conducted on the sine wave path are summarized in Table 2. Please note that when the linear plant model is applied in the 70 km/h scenario, the results are calculated solely for the time interval of 0–4.2 s, since at 4.2 s, the vehicle left the path. In the first scenario, the controller presented in Section 4 is tested, using the vehicle model presented in Section 2.2 for state prediction. The results at 70 km/h vehicle velocity are shown in Figure 16. The controller drives the vehicle accurately and stably, the maximum sideslip angles are 4 deg, and the small values in the lateral and the angular errors also demonstrate the accuracy of the controller.

In the next scenario, the nonlinear vehicle plant model is replaced by a linear one, in which small angle assumptions are applied during the linearization of the model, and the vehicle model is coupled with a linear tire model, while the model of the steering dynamics remains unchanged. The resulting linear vehicle model is described by Equations (51)–(55),

(51) $$V_{y}=\dot{y}$$

(52) $$a_{y}={\dot{V}}_{y}=\frac{-c_{f}v_{y}-c_{f}l_{f}r}{mV_{x}}+\frac{c_{f}\delta }{m}+\frac{-c_{r}v_{y}+c_{r}l_{r}r}{mV_{x}}-V_{x}r$$

(53) $$r=\dot{\varphi }$$

(54) $$\dot{r}=\frac{-l_{f}c_{f}v_{y}-l_{f}^{2}c_{f}r}{I_{z}V_{x}}+\frac{l_{f}c_{f}\delta }{I_{z}}+\frac{l_{r}c_{r}v_{y}-l_{r}^{2}c_{r}r}{I_{z}V_{x}}$$

(55) $${\dot{\delta }}_{act}=-\frac{1}{T_{st}}\delta_{act}+\frac{1}{T_{st}}\delta_{dem}$$

where the lateral force is a linear function of the sideslip angle (56).

(56) $$F_{yf}=-c_{\alpha f}\alpha_{f},\;\;\;F_{yr}=-c_{\alpha r}\alpha_{r\;}$$

In (56), $c_{\alpha f}$ and $c_{\alpha r}$ are the cornering stiffness of the front and rear tires, respectively, which are identified from the measurement results (Figure 2), and the state vector remains unchanged, *x* = [$v\;V_{y}\;\varphi \;r$ $\delta_{act}$]*^T^*.

**Figure 17:**
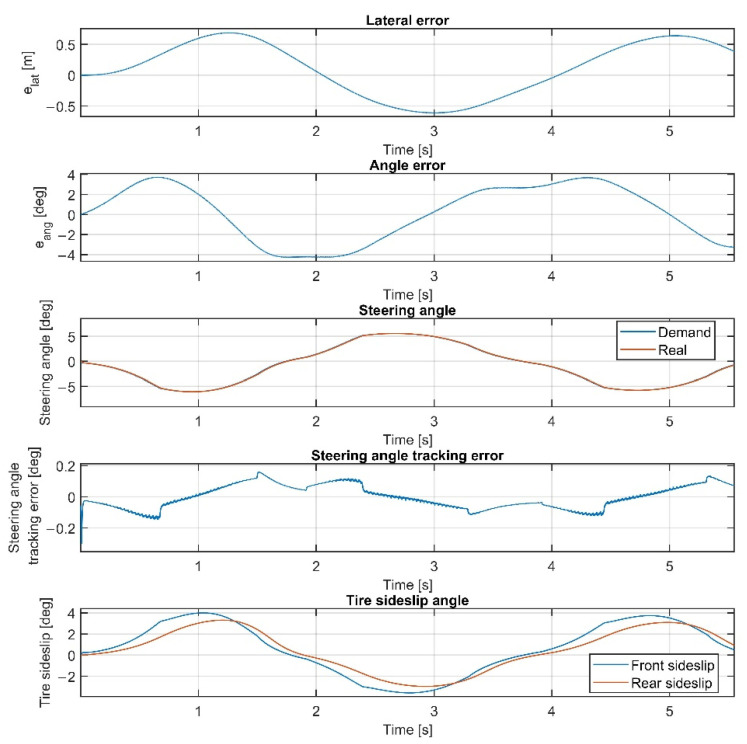
Simulation results on the sine wave path at 60 km/h, including steering dynamics, using the linear vehicle model for state prediction.

**Figure 18:**
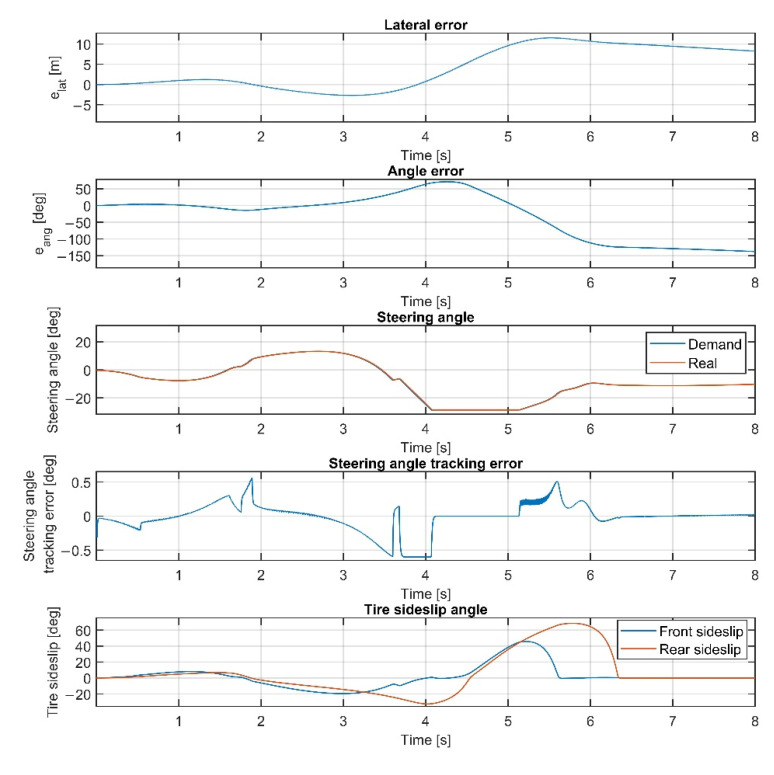
Simulation results on the sine wave path at 70 km/h, including steering dynamics, using linear the vehicle model for state prediction.

The first test using the linear plant model is conducted at 60 km/h and the results are shown in Figure 17. Both the lateral and angular errors are large, while the controller is unable to drive the vehicle along the path when the velocity is increased to 70 km/h, as shown in Figure 18.

In the 70 km/h scenario, the vehicle is unable to follow the path anymore, resulting in leaving the path. The reason for the poor performance of the linear-model-based control is the neglection of the nonlinearities of the controlled vehicle in the state prediction. Both the nonlinear terms in the vehicle model and the tire model are neglected, which results in a less accurate state prediction; hence, in the 60 km/h scenario, performance degradation of the controller is realized, and at 70 km/h, the controller is unable to drive the vehicle along the path.

The results clearly show that the application of the nonlinear vehicle model coupled with the nonlinear tire model for state prediction has a significant advantage over the linear vehicle model and linear tire model couple. Using the nonlinear model, the controller is able to drive the vehicle stable at higher speeds and more accurately than with the linear model. Furthermore, the effectiveness of the proposed LTV-MPC method is also confirmed in this section by testing the controller in the sine wave path.

## 7. Conclusions

In this paper, an LTV-MPC structure is proposed for path-following scenarios. The controller uses a nonlinear vehicle model coupled with a Pacejka tire model for state prediction. Furthermore, the dynamics of the steering system are also incorporated into the prediction model. The reference definition is embedded into the LTV-MPC, corresponding to the states in the plant model, which results in a simplified plant model, removing two equations from the model, which are responsible for the coordinate transformation. The presented reference definition method can reduce the nonlinear terms in the path-following problem definition by transforming the path into the ego frame, resulting in a simplified description of the vehicle dynamics, the nonlinearities of which are easier to handle. Using this method, the transformation from the ego frame to the global frame is practically conducted separately from the vehicle model.

During the simulation tests, the inclusion of the steering dynamics results in a more accurate path-following performance with smaller errors and smoother steering command demanded by the controller. Furthermore, the proposed reference definition is proven to be effective, while the nonlinear terms in the plant model are reduced. The proposed controller structure coupled with the plant model can handle the vehicle at sharp corners when the tires operate in the nonlinear region. The vehicle drives stably during the tests, while the errors remain low. According to the results, the controller is applicable for even emergency scenarios, e.g., for a double-lane change. The effectiveness of the proposed controller is tested in a sine wave path, where the controller is proven to be as accurate as on the double-lane-change path. Furthermore, the effect of the linear and nonlinear plant model is also analyzed and the advantage of applying the nonlinear plant model is clearly proven on the sine wave path.

Regarding future research opportunities, the vehicle plant model and the model of the steering system, as an actuator, could be further detailed, which could result in more accurate state prediction. The dynamics of the steering system could be modeled as a function of the vehicle velocity, the yaw rate, and the lateral acceleration, and the self-aligning torque of the tires could be incorporated into the plant model as well. Furthermore, the proposed reference definition may reduce the computational requirements of an NLMPC, as the plant model has fewer nonlinear terms.

## Figures and Tables

**Table 1:** Simulation results.

Speed [km/h]	Steering Dynamics	e_avg_ [m]	e_max_ [m]	ϕ_avg_ [deg]	ϕ_max_ [deg]
50	No	0.066	0.220	0.516	2.561
	Yes	0.023	0.200	0.510	3.862
60	No	0.085	0.425	0.407	6.159
	Yes	0.077	0.362	0.378	5.363
70	No	0.544	4.672	1.942	25.336
	Yes	0.522	4.669	1.937	25.869

**Table 2:** Simulation results on the sine wave path.

	Speed [km/h]	e_avg_ [m]	e_max_ [m]	ϕ_avg_ [deg]	ϕ_max_ [deg]
Nonlinear plant model	70	0.098	0.192	0.689	2.414
Linear plant model	60	0.390	0.687	2.442	4.259
	70	1.225	2.903	15.307	71.385

## Data Availability

Not applicable.
